# Immunoglobulin G from bovine milk primes intestinal epithelial cells for increased colonization of bifidobacteria

**DOI:** 10.1186/s13568-020-01048-w

**Published:** 2020-06-18

**Authors:** Sinead T. Morrin, Geoffrey McCarthy, Deirdre Kennedy, Mariarosaria Marotta, Jane A. Irwin, Rita M. Hickey

**Affiliations:** 1 Teagasc Food Research Centre, Moorepark, Fermoy, Co. Cork, P61C996 Ireland; 2 Veterinary Sciences Centre, School of Veterinary Medicine, University College Dublin, Belfield, Dublin 4, Ireland

**Keywords:** Immunoglobulin G, Milk, *Bifidobacterium*, Glycosylation, Adhesion

## Abstract

A bovine colostrum fraction (BCF) was recently shown to enhance the adherence of several commensal organisms to intestinal epithelial cells through modulating the epithelial cell surface. In this study, the main components of the BCF were examined to investigate the active component/s responsible for driving the changes in the intestinal cells. The adherence of various bifidobacteria to HT-29 cells was increased when the intestinal cells were pre-incubated with immunoglobulin G (IgG). Modulation of the intestinal cells by IgG was concentration dependent with 16 mg/mL IgG resulting in a 43-fold increase in the adhesion of *Bifidobacterium longum* NCIMB 8809 to HT-29 cells. Periodate treatment of colostral IgG prior to performing the colonization studies resulted in a reduction in the adhesion of the strain to the intestinal cells demonstrating that the glycans of IgG may be important in modulating the intestinal cells for enhanced commensal adhesion. IgG isolated from mature milk also resulted in significant increases in adhesion of the *Bifidobacterium* strains tested albeit at reduced levels (3.9-fold). The impact of IgG on the HT-29 cells was also visualised via scanning electron microscopy. This study builds a strong case for the inclusion of IgG ingredients sourced from cow’s milk in functional foods aimed at increasing numbers of health promoting bacteria in the human gut.

## Key points

- IgG from milk modulates intestinal cells increasing the adherence bifidobacteria.
- The bioactivity of IgG is concentration dependent.
- The glycans of IgG are involved in modulating the intestinal cells.
- IgG sourced from cow’s milk has potential as a functional food ingredient.

## Introduction

Bifidobacteria are among the first bacteria to colonize the human gastrointestinal tract and are believed to confer beneficial health benefits on their host (O’Callaghan and van Sinderen 2016; Plaza-Diaz et al. 2018). These bacteria exhibit health-promoting or probiotic effects such as modulation of the immune system (O’Hara and Shanahan 2007), protection of the host against pathogens by competitive exclusion (Bernet et al. 1994; Hooper et al. 1999) and provision of nutrients through the degradation of non-digestible dietary carbohydrates (Roberfroid et al. 1995; Leahy et al. 2005). Bifidobacteria not only need to survive GI transit, they also need to colonize the GI tract. An effective probiotic must reside at the preferred target sites in the GI tract long enough and at adequate concentrations to confer probiotic effects. There are currently no commercial products available that aid or increase the attachment of health-promoting bacteria to the gut mucosal surface despite the growing market for probiotics.

The ingestion of colostrum and milk may be expected to have the greatest influence on microbial colonization in the early stages of life. Milk not only helps to inoculate the infant with a milk associated microbiota (Jost et al. 2013), but colostrum, the first milk, may be expected to have the greatest effect on the intestinal epithelium. The milk secreted by cows during the first few days after parturition is known as colostrum. It contains many essential nutrients and bioactive constituents, including immunoglobulins, growth factors, α-lactalbumin (α-LA) and β-lactoglobulin (β-lg), lysozyme, lactoperoxidase, lactoferrin (LF), nucleosides, cytokines, vitamins, peptides and oligosaccharides, all of which are relevant to gut health (Pecka-Kielb et al. 2018). It should be noted however, that β-lg is not found in human milk (Brignon et al. 1985). In certain parts of the world, bovine colostrum is used as a food supplement since many studies have indicated that it may have anti-atherosclerotic, anti-cancer, anti-bacterial and anti-oxidant properties (Godhia and Patel 2013). Importantly, it can stimulate the development and function of the gastrointestinal tract (Hadorn et al. 1997; Guilloteau et al.^1997^; Blum and Hammon, 2000a, b). Studies have shown the potential of colostrum in restoring the composition of the intestinal microbiome and in supporting the healing of damaged mucosa (Champagne et al. 2014; Playford et al. 1999; Cairangzhuoma et al. 2013) and may in part explain its growing popularity as a supplement aimed at improving gut health. For instance, bovine colostrum has been shown to increase the activity of specific brush-border enzymes and decrease severity of necrotising enterocolitis when compared with feeding milk formula in preterm born piglets (Støy et al. 2014). In term-born piglets, bovine colostrum reduced the intestinal colonisation of enterotoxigenic *Escherichia coli* unlike milk replacer (Sugiharto et al. 2015). Poulsen et al. (2017) demonstrated that there was a higher abundance of LAB genera (for example, *Lactococcus* and *Leuconostoc*) observed in bovine colostrum-fed piglets when compared with milk replacer-fed piglets. Another recent study demonstrated its effectiveness in decreasing intestinal permeability in athletes during peak training for competition (Halasa et al. 2017). While these studies demonstrate bovine colostrum can modulate the intestinal epithelium and the gut microbiota profile, the exact mechanisms by which these changes occur and the individual milk components responsible remain largely unknown.

We recently demonstrated via in vitro assays that bovine colostrum modifies the intestinal cell surface, and in turn the attachment of bifidobacteria and lactobacilli (Morrin et al. 2019a). In the study, human HT-29 intestinal cells were exposed to a bovine colostrum fraction (BCF) which resulted in an up to 52-fold increase in bacterial attachment to the mammalian cells. The fact that exposure of the HT-29 cells to the BCF resulted in increased adherence of all bifidobacterial strains tested and a commercial probiotic *Lactobacillus* strain is of particular interest. Importantly, alteration of the HT-29 cell surface did not result in increased adhesion of the enteric pathogens tested (Morrin et al. 2019a). Analysis of the transcriptome, proteome and glycome of the intestinal cells following treatment with BCF indicated that the cell surface was being modulated through changes in the expression levels of certain glycoproteins and through enzymatic addition of glycans to glycoconjugates (Morrin et al. 2019a, b). In the current study, we aim to investigate the milk component/s in the BCF responsible for promoting the adherence of commensal bacteria to the human intestinal cells. Compositional analysis of the BCF was performed and the individual components present in the BCF were examined for their effect on the HT-29 cells and in turn bifidobacterial adhesion.

## Materials and methods

### Materials

The oligosaccharide standards, 3′-siallyllactose (3′-SL) and 6′-sialyllactose (6′-SL) were purchased from Carbosynth Ltd (Berkshire, UK). Bovine milk derived α-LA and β-lg were supplied by Merck (Darmstadt, Germany).

### Isolation of bovine colostrum fraction (BCF)

Bovine colostrum (Day 1) and mature milk were collected from Holstein–Friesian cattle on-site at Teagasc Food Research Centre, Moorepark (Fermoy, Cork, Ireland) and underwent fat and casein removal using conventional methods as described by Kobata et al. (1969). In brief, defatting was performed through centrifugation and then caseins were precipitated under acidic conditions (1 M HCl) and then removed through centrifugation. Many of the larger peptides were reduced from the resulting supernatant by ultrafiltration and lactose was reduced using a Sephadex G-25 column (Pharmacia, Uppsala, Sweden). The bovine colostrum fraction was isolated using High pH Anion Exchange Chromatography with Pulsed Amperometric Detection as previously described by Morrin et al. (2019a).

### SDS-PAGE analysis of the BCF

Sample preparation and reduction for sodium dodecyl sulphate polyacrylamide gel electrophoresis (SDS-PAGE) analysis was performed as per manufacturer’s instructions (BioRad Laboratories Ltd., Hertfordshire, UK). The BCF were separated on a 4–12% pre-cast polyacrylamide gel (BioRad Laboratories Ltd., Hertfordshire, UK) using 13 µL of sample (containing 10 μg of protein). Protein bands were visualized on the gels using Coomassie blue stain (Merck) following the manufacturer’s procedure.

### Compositional analysis of the BCF

The oligosaccharide content of the BCF was estimated using High pH Anion Exchange Chromatography with Pulsed Amperometric Detection (HPAEC-PAD) as per Morrin et al. (2019a). Samples were separated and quantified for lactose using an Aminex HPX 87C Carbohydrate column (30,067.8 mm) (Bio-Rad, UK) fixed ion resin column as per Morrin et al. (2019a). Levels of β-lactoglobulin (β-lg) and α-lactalbumin (α-LA) were determined using a TSK G2000SW (300 × 7.5 mm) and a TSK G2000swxl column (300 × 7.8 mm, from Tosu Hass, Japan) linked in series and fitted to a Waters Alliance 2695 separation module (Waters Corporation, Milford, Mass, USA) as per Morrin et al. (2019a). Levels of LF, immunoglobulin G (IgG) and A (IgA), were determined using ELISA quantification kits (Bethyl Laboratories, Inc. Cambridge Bioscience, Cambridge, UK) as per Morrin et al. (2019a).

### Isolation of oligosaccharides and lactose from the BCF

The BCF (4 mg/mL) was fractionated by centrifugal filtration using a 3 kDa MWCO (Merck Millipore, Cork, Ireland). HPAEC-PAD analysis, as described above, confirmed the permeate contained the oligosaccharides and lactose while the retentate contained proteins and peptides greater than 3 kDa (confirmed by HPLC, previously described above). The permeate was examined in the adhesion assay described below.

### Isolation of IgG from bovine colostrum and milk

Day 1 colostrum and mature milk samples were pooled separately and defatted and decaseinated as described by Kobata et al. (1969). Due to the relatively low levels of IgG in mature milk, an ultrafiltration (UF) step was employed using a 50 kDa molecular weight cut-off, spiral-wound, polymeric membrane at 50 °C (Synder Membrane Technology Co., Ltd., Vacaville, USA). Confirmed via the quantification methods mentioned previously, this step resulted in higher transmission levels of IgG from the mature milk fraction, prior to its subsequent purification. Soluble IgG was isolated from the pooled skimmed bovine colostrum and mature milk using a 5 mL HiTrap Protein G column, Sepharose HP as per manufacturer’s instructions (GE Healthcare, Little Chalfont, UK). The skimmed colostrum was diluted in dH_2_O to give 2% (w/v) total solids and further diluted 1:1 in binding buffer (20 mM phosphate buffer, pH 7.4) to give a concentration of 11 mg/mL. The skimmed mature milk was freeze dried and resuspended in binding buffer (20 mM phosphate buffer, pH 7.4) to give a concentration of 32 mg/mL. Separately, both samples were loaded on to the column which was equilibrated with the binding buffer at a flow rate of 4 mL/min. The column was washed with 5 column volumes (25 mL) of binding buffer and IgG was eluted with elution buffer (100 mM glycine–HCl, pH 2.7). The recovered IgG-containing fractions were immediately neutralized with 1 M Tris⁄HCl (pH 9) and the protein concentration was determined using the Quick Start Bovine γ-Globulin Standard (BioRad Laboratories Ltd.). Fractions with the highest concentration of IgG from each run were pooled. The protein profile of the sample at each step was analysed by reducing SDS-PAGE as described above to confirm the purity of pooled IgG sample. The purified IgG samples were then freeze-dried and stored at 4 °C.

### Bacterial strains growth conditions

*Bifidobacterium longum* subsp. *infantis* (ATCC^®^ 15697™), *Bifidobacterium longum* NCIMB 8809 and *Bifidobacterium breve* NCFB 2258 were obtained from the American Type Culture Collection (ATCC, Middlesex, UK), the National Collection of Industrial and Marine Bacteria (Aberdeen, Scotland, UK) and the National Collection of Food Bacteria at the AFRC Institute of Food Research (Reading, UK) respectively. The strains were stored in deMan Rogosa Sharpe (MRS) (Difco, Sparks, MD, USA) broth containing 50% glycerol at − 80 °C until they were re-cultured according to the supplier’s instructions. The strains were cultured at 37 °C under anaerobic conditions generated using an Anaerocult A system (Merck). To prepare mid-exponential phase cells, overnight cultures were adjusted to an optical density of 0.3 and the cultures were then grown for a further 2 h and then adjusted to an optical density (OD_600nm_) of 0.45–0.5.

### Epithelial cell line conditions

The human colon adenocarcinoma cell line HT-29 was purchased from the American Type Culture Collection (ATCC). HT-29 cells were routinely grown in McCoy’s 5A modified medium (Merck). All cells were routinely maintained in 75 cm^2^ tissue culture flasks and incubated at 37 °C in a humidified atmosphere (5% CO_2_). Cells were passaged when the confluency of the flasks reached approximately 80%. HT-29 cells were seeded into 12 well plates (Corning^®^, NY, USA) at a concentration of 1 × 10^5^ cells/well and incubated at 37 °C in 5% CO_2_ in a humidified atmosphere. The cells were fed every second day with McCoy’s media (10%, v/v, FBS (Merck)) until 100% confluency was reached.

### Cell culture and exposure of HT-29 cells to individual BCF components

Prior to sample exposure, the cells were washed and placed in McCoy’s 5A modified medium supplemented with 2% (v/v) FBS. After 24 h, the cells were washed with phosphate buffered saline and treated with filter-sterilised milk components (described below) which had been re-suspended in pre-heated un-supplemented McCoy’s 5A modified medium. Non-supplemented McCoy’s 5A medium was used as a non-treated control (NT). The cells were then washed with phosphate buffered saline and the treatments (described below) were applied. The plates were then incubated at 37 °C for 24 h in a humidified atmosphere (5% CO_2_) prior to bacterial exposure. The HT-29 cells were exposed to the following treatments: Oligosaccharides and lactose (permeate of 3 kDa cut-off of BCF, total concentration of 4 mg/mL), α-LA (8 mg/mL), β-lg (8 mg/mL) and IgG (24 mg/mL). To determine if IgG concentration influenced the HT-29 cells and in turn the adherence of commensal bacteria, the adhesion assay was repeated as described above using IgG at the following concentrations: 2, 6, 8, 12, 16 and 24 mg/mL. To determine if IgG isolated from mature milk influenced the HT-29 cell surface and subsequently commensal adherence, the adhesion assay was once again repeated using mature milk derived IgG. Mature IgG was tested at a concentration of 16 mg/mL corresponding to the optimum concentration revealed in the concentration dependency assay of colostral IgG. All experiments were performed in triplicate. Before bacterial exposure, the HT-29 cells were washed three times with PBS to remove residual milk components.

### Adhesion assay

An overnight culture of either *Bifidobacterium longum* subsp. *infantis* ATCC^®^ 15697™, *Bifidobacterium longum* NCIMB 8809 or *Bifidobacterium breve* NCFB 2258ATCC 15697 was adjusted to an optical density (OD_600nm_) of between 0.45 and 0.5 by centrifugation (3920*g* for 10 min) as per Kavanaugh et al. (2013). The bacterial cells were washed twice in un-supplemented McCoy’s 5A modified medium before re-suspension in the same media. The bacterial cells were then exposed to the treated intestinal cells for 2 h at 37 °C under anaerobic conditions using an Anaerocult A system (Merck). Exposure conditions were adjusted before the experiment to maintain maximal viability of bacterial and HT-29 cells. The wells were then washed four times with PBS to remove non-adherent bacteria and lysed with 500 µl/well of 0.1% Triton X100 (Merck) for 10 min at 37 °C. The lysates were serially diluted and bacteria enumerated by spread-plating on MRS plates. The plates were incubated anaerobically at 37 °C for 48 h and CFU were then counted. Adhesion was determined as the fold change difference between the bacteria attached to the control (non-treated, NT) HT-29 cells and bacteria attached to the treated HT-29 cells. Fold adhesion = [CFU/mL of recovered adherent bacteria on treated HT-29 cells/CFU/mL of recovered adherent bacteria on control (NT) HT-29 cells]. Adhesion assays were performed in triplicate over 3 successive passages of intestinal cells.

### Sodium metaperiodate treatment of colostral IgG

Sodium metaperiodate treatment of colostral IgG was performed as previously described by Alemka et al. (2010). IgG (24 mg/mL) was incubated with 0.011 mM sodium metaperiodate (Merck) dissolved in PBS. The mixture was then incubated at room temperature for 30 min. Excess sodium metaperiodate was removed by centrifugal filtration using a 3-kDa molecular weight cut-off with 3 × 1 mL PBS, pH 7.4, washes and the retentate containing meta-periodate treated IgG was diluted to its original volume and stored at − 20 °C. The adhesion assay was then carried out with the metaperiodate-treated IgG and *B. longum* 8809 as described above.

### Scanning electron microscopy of *Bifidobacterium longum* NCIMB 8809 adhesion to IgG-treated HT-29 cells

Scanning electron microscopy (SEM) was employed to visualise the attachment of *Bifidobacterium* cells to IgG treated HT-29 cells versus untreated HT-29 cells. For adhesion assays, HT-29 cells (approximately 1 × 10^5^ cells/mL) were seeded in 12-well tissue culture plates (Merck) on microscopy cover glasses in McCoy’s 5A modified medium (Merck) and incubated at 37 °C in a humidified atmosphere of 5% CO2, until reaching 90 to 100% confluency. HT-29 cells were exposed to 16 mg/mL colostral IgG as described previously. Non-supplemented McCoy’s 5A medium was used as a non-treated control (NT). The adhesion assay was performed as previously described above. After 2 h of incubation with *B. longum* 8809, the cells were then observed by Scanning Electron Microscopy (SEM) using the method of Khokhlov et al. (2012). The HT-29 monolayer was washed three times with 1 mL PBS and fixed with 4% (w/v) glutaraldehyde (Merck), in 0.1 M phosphate (PB) buffer, (pH 6.8–7.4) for 1 h at room temperature. After washing three times with PBS, the HT-29 monolayer was dehydrated in a graded ethanol series (25% v/v, 50% v/v, 75% v/v, 95% v/v and 100% v/v). The cells were air dried and sputter coated with chromium gold (Emitech K550X, Ashford, UK). The microscope cover glasses were then examined with a Field Emission Scanning Electron Microscope at 7 kV (FE-SEM; Zeiss Supra Gemini, Darmstadt, Germany).

## Results

### Compositional analysis of the BCF

Compositional analysis revealed that the BCF contained 1.68 mg/mL lactose (42% of total solids TS), 0.56 mg/mL sialyllactose (3′SL and 6′SL, 14% TS), 8 μg/mL α-LA and 8.1 μg/mL β-lg. Further analysis via ELISA demonstrated that the BCF contained 24 μg/mL IgG, 0.04 μg/mL IgA and trace amounts of lactoferrin (1.92 × 10^−6^ mg/mL). Most likely‚ much of the remaining TS in the BCF consists of inorganic mineral matter (ash) which is present in bovine milk at levels up to 7–8 g/L (Luce and Horne 2009). Indeed, the membrane filtration step would have concentrated levels of ash, organic acids and non-protein nitrogen present in the BCF. SDS-PAGE analysis of the BCF revealed the presence of four main protein bands at 13 kDa, 16 kDa, 25 kDa and 50 kDa (data not shown). Considering the HPLC and ELISA results in combination with the SDS-PAGE, it was concluded that the main components of the BCF included α-LA, β-lg, IgG, oligosaccharides and lactose.

### Identification of active component/s in the BCF

Increases in adhesion of *Bifidobacterium longum* subsp. *infantis* ATCC 15697 to HT-29 cells after prior exposure to the major components of BCF were investigated (Table 1). The oligosaccharide and lactose fraction (MW ~ 300–1200 Da) present in the 3 kDa permeate of the BCF did not result in an increase in adhesion of *B. longum* subsp. *infantis* to the HT-29 cells (p < 0.05). Although the BCF was found to contain IgG, α-LA and β-lg in µg/mL quantities, we increased the concentration used in the assays to mg/mL (24, 8 and 8 mg/mL respectively) to be more reflective of physiological concentrations found in milk. As the BCF underwent membrane filtration, the proteins in the sample may be fragments of the intact proteins which may still be detected by the ELISAs. For this reason, it would have been impossible to replicate the status of the proteins/peptides in the BCF for the purposes of the bioassays and therefore concentrations were increased 1000-fold. Each protein was examined individually for their ability to increase the adhesion of the *B. infantis* 15697 to the HT-29 cells (p < 0.05). While α-LA and β-lg at these concentrations had little effect on adhesion, IgG resulted in dramatic 9.2-fold increase in the adhesion of the strain (p < 0.05).

**Table 1:** Adhesion of *Bifidobacterium longum* subsp. *infantis* 15697 to HT-29 cells treated with the various components present in the BCF

	Fold change	*p* value
Lactose and Oligosaccharides	0.4	3.5 × 10^−10^
IgG (24 mg/mL)	9.2	9.7 × 10^−4^
α-lactalbumin (8 mg/mL)	0.4	2.2 × 10^−7^
β-lactoglobulin (8 mg/mL)	1.3	7.2 × 10^−3^

### IgG primes intestinal epithelial cells for increased colonization of bifidobacteria

**Fig. 1:**
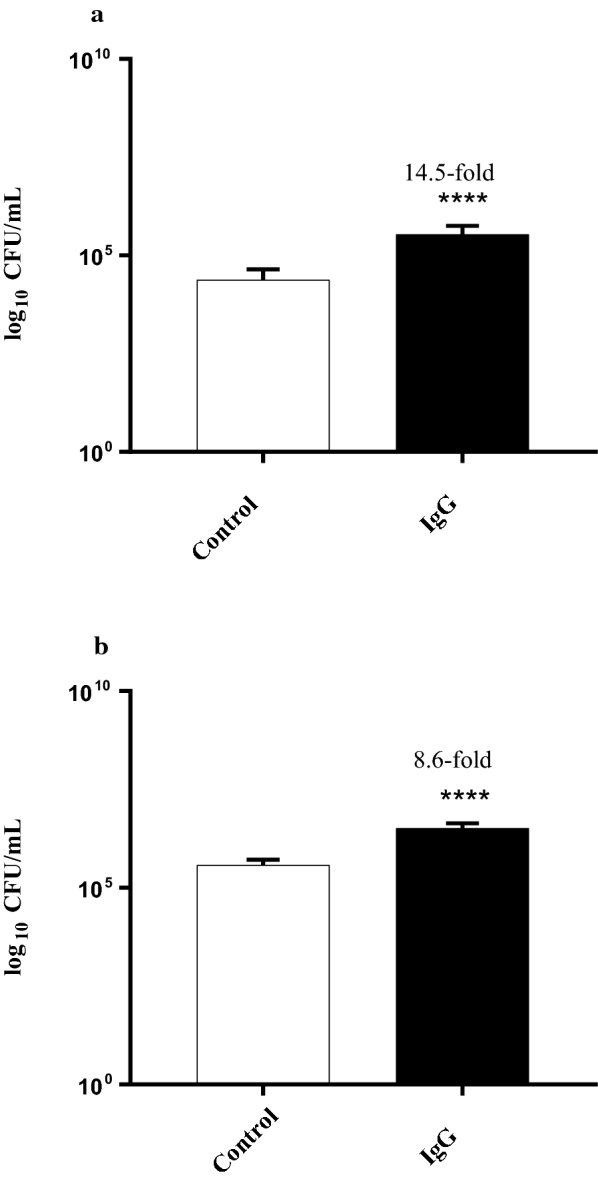
Adhesion of **(a)***Bifidobacterium longum* NCIMB 8809 and **(b)***Bifidobacterium longum* subsp. *infantis* 2258 to IgG treated HT-29 cells. *Denotes a significant difference compared to the control, where p < 0.05

**Fig. 2:**
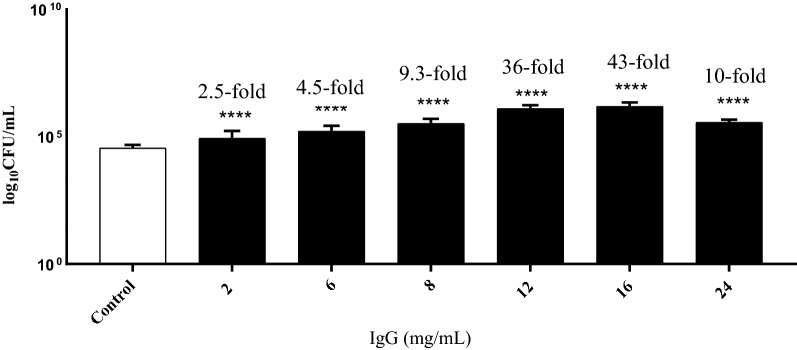
*Bifidobacterium longum* NCIMB 8809 adherence to HT-29 cells treated with varying concentrations of purified IgG. *Denotes a significant difference compared to the control where *p* < 0.05. Fold changes over onefold are denoted

Two further *Bifidobacterium* sp. were then examined for their adherence to HT-29 cells that had been exposed to 24 mg/mL of IgG (Fig. 1). *Bifidobacterium longum* subsp. *longum* NCIMB 8809 and *Bifidobacterium breve* 2258 displayed a 14.5-fold and a 8.6-fold increase in adhesion (p < 0.05) respectively suggesting that IgG or fragments thereof in the BCF was responsible for the adherent phenotype of the HT-29 cells. To examine the effect of IgG concentration on the HT-29 cells, the cells were exposed to IgG concentrations ranging from 2 to 24 mg/mL (Fig. 2). The highest increase (43-fold, p < 0.05) in adherence of *Bifidobacterium longum* subsp. *longum* NCIMB 8809 to the cells was observed after exposure to 16 mg/mL IgG. The fold change in adhesion decreased after the 16 mg/mL IgG exposure, indicating that this concentration was the optimum for bioactivity.

**Fig. 3:**
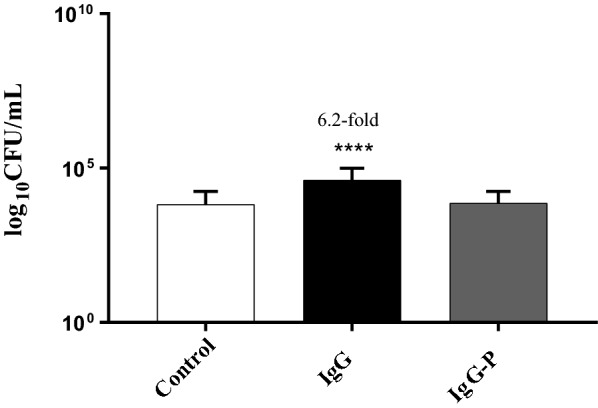
*Bifidobacterium longum* NCIMB 8809 adherence to HT-29 cells exposed to sodium metaperiodate-treated IgG and untreated IgG. *Denotes a significant difference compared to the control where *p* < 0.05. Fold changes over onefold are denoted

**Fig. 4:**
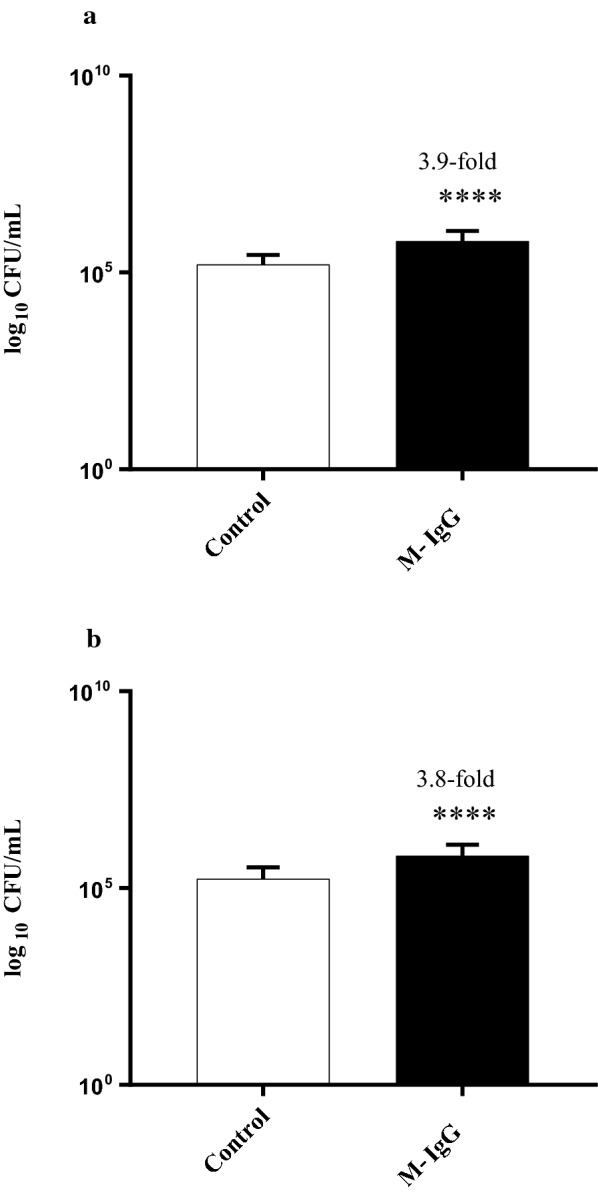
Adhesion of **(a)***Bifidobacterium longum* NCIMB 8809 and **(b)***Bifidobacterium longum* subsp. *infantis* 15697 to mature milk derived IgG (M-IgG) treated HT-29 cells. *Denotes a significant difference compared to the control, where p < 0.05

The HT-29 cells were then exposed to sodium metaperiodate-treated IgG and the adhesion assay was performed with the *B. longum* strain. No significant increase in adherence was observed in cells exposed to periodate treated IgG (Fig. 3). Periodate oxidation of glycoproteins converts vicinal diols of oligosaccharide residues to 2 aldehyde groups, making the carbohydrate structures biologically unrecognizable. This result suggests that IgG glycosylation plays a very important role in driving the HT-29 adherent phenotype. Exposure of HT-29 cells to IgG isolated from mature bovine milk (Fig. 4) also resulted in a significant increase in adhesion of *B. longum* NCIMB 8809 (3.9-fold, p < 0.05) and *B. longum* subsp *infantis* ATCC 15697 (3.8-fold, p < 0.05). The increase in the adhesion of both strains was significantly lower when compared to colostral IgG treated HT-29 cells, suggesting that changes in IgG across lactation such as variations in glycosylation influence the bioactivity.

**Fig. 5:**
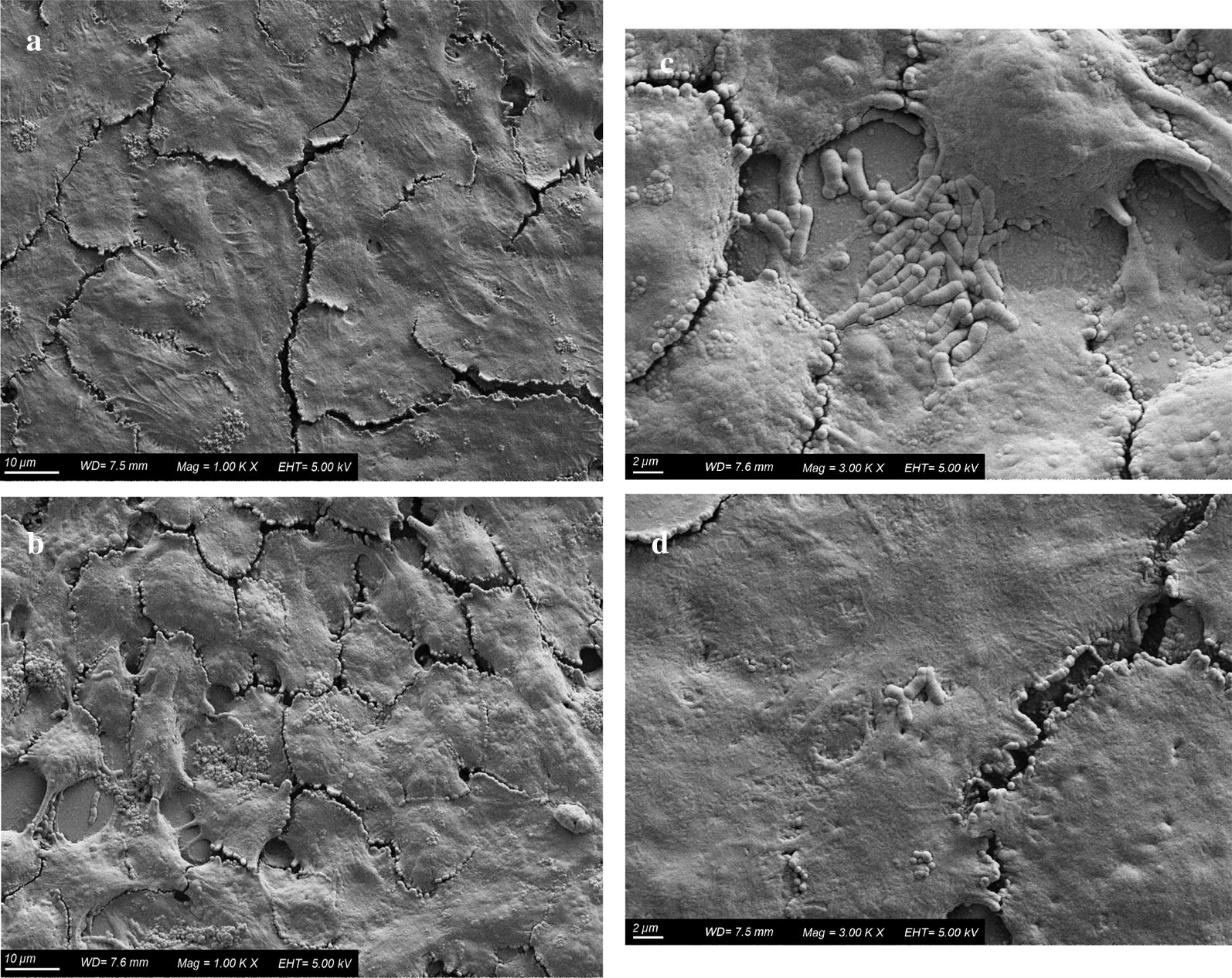
Monolayer of (**a**) non-treated and (**b**) IgG-treated HT-29 colonic epithelial cells with *Bifidobacterium longum* NCIMB 8809 (10 μm, magnification 1000 ×). Monolayer of (**c**) IgG-treated and (**d**) non-treated HT-29 colonic epithelial cells with *Bifidobacterium longum* NCIMB 8809 (2 μm, magnification 3000 ×)

Scanning electron microscopy of cells exposed to IgG (Fig. 5) revealed phenotypic differences compared to the control (non-treated) HT-29 cells with the surface of the IgG treated cells appearing smoother. Examination of *B. longum* NCIMB 8809 adhesion to control and IgG treated HT-29 cells was also visualised by SEM (Fig. 5). The attachment of higher number of *Bifidobacterium* cells to IgG treated HT-29 cells was evident when compared to the non-treated control.

## Discussion

Little information exists regarding the direct interaction of colostrum with the gastrointestinal surface and the ensuing effect this has on the colonisation of bacteria. Previously, exposure of BCF to HT-29 colonic epithelial cells led to a significant increase in adhesion of several *Bifidobacterium* strains (Morrin et al. 2019a). The BCF was found to modify the glycosylation pattern of the cell surface as confirmed by gene microarray, proteomic and lectin array analysis (Morrin et al. 2019a, 2019b). In the current study, we aimed to explore which component/s of the BCF was responsible for this bioactivity. Characterisation of the BCF revealed that low levels of protein were present. This is expected as the fraction underwent removal of large whey proteins such as caseins and fat and had also been passed through a G-25 Sephadex column (Morrin et al. 2019a). The analysis revealed that α-LA, β-lg, IgG or fragments thereof, oligosaccharides and lactose were the major components of the BCF and screening of these components for their effect on HT-29 cells and subsequent adhesion of bifidobacteria was the focus of this study.

The fraction of the BCF containing the oligosaccharides and lactose had no significant effect on bacterial adhesion when tested in this study. Similarly, α-LA and β-lg had little effect on the numbers of bifidobacteria adhering to the cells. IgG is the dominant Ig in colostrum and bovine milk and makes up 80–90% of the total Ig in colostrum (Stelwagen et al. 2009). IgG is known for its potent immunological properties and its ability to deter gastrointestinal pathogens such as bacteria, protozoa and viruses (Ellens et al. 1978; Rump et al. 1992; Lissner et al. 1996; den Hartog et al. 2014). Three *Bifidobacterium* strains, previously shown (Morrin et al. 2019a) to adhere in greater numbers to HT-29 cells after exposure to BCF (*B. infantis* 15697 *B. longum* 8809 and *B. breve* 2258), were examined with IgG-treated HT-29 cells and a significant increase in the adhesion of all three strains was observed. The effect of IgG on the HT-29 cells was concentration dependent with 16 mg/mL being the optimal concentration for increasing adherence of the bacteria. The effect of IgG (43-fold increase) at this concentration was similar to that of the original BCF which resulted in a 52-fold increase in *B. longum* adhesion to the cells (Morrin et al. 2019a).

IgG is heavily glycosylated and the intact IgG glycoprotein consists of 90% protein and 10% carbohydrate (El-Loly 2007) with *N*-glycosylation conserved at the Asn297 site of the Fc region (Takimori et al. 2011; Recio et al. 2009). In this study, IgG was treated with sodium metaperiodate, which oxidises and opens saccharide rings between vicinal diols. Exposure of HT-29 s cells to periodate-treated IgG did not result in a significant increase in adhesion of the *B. longum* 8809 strain. This indicates that the glycan moieties of IgG may be largely responsible for the observed increase in bifidobacterial adhesion. Glycosylation of IgG is also known to vary over lactation (Takimori et al. 2011; Feeney et al. 2019). Interestingly, exposure of the HT-29 cells to mature IgG led to a significant increase in adhesion of *Bifidobacterium*, albeit at a much lower level when compared to the colostral IgG. As the observed effect of IgG in this study is potentially linked to its carbohydrate portion, the glycosylation pattern of IgG over lactation may directly correlate to the differences observed in adhesion between the two variants. The fact that IgG derived from mature bovine retains activity is of particular interest considering the commercial relevance of isolating bioactive IgG from mature milk and whey streams.

SEM images indicated treatment of the HT-29 epithelial cell surface with both IgG and the *Bifidobacterium* strain resulted in a smoother surface and higher adherence of the strain compared to their non-treated counterparts. The smoother surface may be accredited to the treatment of the cell surface with either IgG or *Bifidobacterium* or both. Numerous studies have demonstrated the beneficial effects of dietary components (Forgie et al. 2019; Zhao et al. 2019) and probiotics (El Aidy et al. 2013; Kurose et al. 2019; O’Connell Motherway et al. 2019) on intestinal cell integrity.

The effects of processing on IgG must also be considered as the IgG described here is isolated from raw colostrum and milk with minimal processing. Exposure of IgG to heat treatments can directly influence its conformation, which in turn may alter its functionality. Many authors have suggested that heat treatment should be kept to a minimum when manufacturing Ig-based functional foods (Bogahawaththa et al. 2017; Gapper et al. 2007; Hurley and Theil 2013). Studies have shown heat treatments of above 65 °C affect the structure of Ig and its functions (Calmettes et al. 1991; Gapper et al. 2007; Li et al. 2005; Dominguez et al. 1997, 2001).

Another factor to consider in utilising IgG as food ingredient is the effect of digestion on the protein and whether it needs to survive passage through the stomach to reach the intestine. A number of studies examining the digestion of orally administered IgG in both infants and adults have demonstrated recovery of intact IgG in the faecal matter to be variable ranging between 0.01 and 50% (Ulfman et al. 2018). The glycosylation of IgG may also enhance passage through the stomach undigested as witnessed with other milk glycoproteins (Boutrou et al. 2008). In the current study, a higher concentration of purified IgG resulted in the optimum bioactivity in terms of bacterial adhesion when compared to the amount of IgG detected in the original BCF. However, it should be noted that the BCF underwent a membrane filtration step before purification (Morrin et al. 2019a) and such processing may result in IgG fragments rather than intact glycoprotein. IgG can be cleaved into F(ab’)2, Fab and Fc fragments (Marnila and Korhonen 2002). These fragments have also been detected in the stool of infants and adults and retain bioactivity (Zeece et al. 2008; López-Expósito et al. 2012). The IgG present in the original BCF may only represent a portion of IgG or may comprise a glycopeptide which may indeed confer enhanced bioactivity.

There are currently no commercial products available that aid or increase the attachment of health-promoting bacteria to intestinal cells. This crucial step in probiotic applications has been largely overlooked. In this context, a new player, represented by milk-derived IgG, may present a means by which the maximum benefit from consumption of probiotics can be achieved through influencing probiotic colonization in the gut. Understanding the influence of IgG on host-microbial interactions at the molecular level, in terms of both host glycan structures and bacterial adhesion capabilities, will be paramount for future applications. Further studies are required to elucidate the exact sugars and monosaccharides of IgG which are acting on the intestinal cells. Overall, this study highlights the potential of bovine IgG as a nutraceutical for conditioning the intestine to reinstate health-promoting bacteria in subjects with reduced levels of these microbes as observed with various gastrointestinal disorders including inflammatory bowel disease, obesity and metabolic syndrome.

## Data Availability

All relevant data are within the manuscript.
